# Diseases of Children

**Published:** 1886-10-23

**Authors:** 


### TILL THE DOCTOR COMES.

[Inquiries and Communications for this column should be addressed, "Emergencies," care of the Editor of THE HOSPITAL.]

### DISEASES OF CHILDREN.—II.

**Feverishness.** So excitable is their nervous system, that the least thing may throw children into a state of violent fever—even some undigested food may do this: so that it is not wise to be unduly alarmed, unless other symptoms declare themselves that point unmistakably to disorder of some particular organ. If indigestion is supposed to be the cause, and the child is quite an infant, a small dose of grey powder can do no harm, and may give relief when it has acted. It is astonishing how small a dose will do good; half a grain is ample, and will act as well as a much larger dose. It may be given in a little milk, and the child will not notice it. Whilst on the subject of medicines, the old practice of giving large nauseous powders may be emphatically condemned: the dose of grey powder suggested, or, for an older child, the same dose of calomel, is ample, quite tasteless, and may be given in a little milk without the child knowing it. These powders, unfortunately, must not be given regularly in obstinate constipation, or they would in time cause serious symptoms. Small pills containing these ingredients (and many other sorts also) are now made up and sold as "parvules." They look like small rose-coloured pills, have a sweetish taste, and children suck them like sweets; they cannot be too highly recommended and used. Further treatment for feverishness may consist of a warm bath, or sponging with warm water, and rest in bed in a well-ventilated, darkened room.

**Teething.** This process re-acts on the nervous system in various ways in different children: one child may have diarrhœa and symptoms of bowel disorder at the cutting of each series of teeth, another will have all the symptoms of a bad cold, whilst another may have a somewhat severe attack of bronchitis at these times. More care must be given to the diet and nursing, and the various disorders must be treated medically. If, as in the more favourable cases, there is only some slight fever, it may be treated as in the former paragraph. Lancing the gums is occasionally useful, but should not be performed till the tooth is evidently just ready to come through, and is giving rise to symptoms of irritability. There is no doubt that teething may give rise to convulsions.

**The First Teeth.** The first teeth generally cut are the two front teeth of the lower jaw (as a rule, those of the lower jaw just precede the upper), then the corresponding teeth of the upper jaw; next will come the four teeth on each side of these. The front teeth usually appear at the seventh month, and the second at the ninth, but there are many exceptions; some children do not begin to cut their teeth till they are a year old. The first set of teeth are generally complete from the age of two years to two and a half. The number of the first set is twenty.

**Thrush.** Thrush generally denotes a weak state of health, and is due to improper food. The great mistake is to begin farinaceous food too soon. This should never be commenced till the child is six months old, except under medical advice, and even then some of the partially malted foods should be used, beginning with very small quantities, as Dr. Mellin's or Liebig's food for infants. It is customary to treat this complaint by smearing the tongue with a nasty mess of borax and honey. As it is due to the development of a fungoid growth, it is much more rational and effectual to keep the mouth frequently wiped with a rag dipped in sanitas lotion, or in a saturated solution of boracic acid. Great attention should always be paid to the cleanliness of feeding-bottles, etc.

**Discharge from the Eyes.** Many new-born children are subject to a discharge from the eyes, which, if neglected, gets very profuse, gumming the lids together after sleep, and soon destroying the eye. It is generally the custom to regard this very lightly, and we mention the subject here to condemn such negligence most emphatically. If treated early the discharge is easily cured, but numbers of eyes are lost annually from neglecting the first stages. Alum lotion (a teaspoonful to a pint of rain water) should be thoroughly washed over the surface of the globe of the eye every half-hour. Wipe all discharge away with a piece of rag dipped in the same lotion, and burn the rag immediately, as the discharge is very infectious. For the same reason, wash the hands well after each application of the lotion, use separate towels, etc.

**Eczema of Head.** This is another disease often much neglected till great mischief is done. If slight, soften the scabs well with plenty of sweet oil, almost continuously applied, and then rub in some vaseline. If severe, the scabs must be softened by using linseed poultices with sweet oil, till they can be removed, and then zinc ointment or vaseline may be used. The general health may require treatment at the same time.

---

DRUGGISTS.—A druggist is not inappropriately termed the pill-ar of society.

